# Genetic Diversity of SARS-CoV-2 in Kazakhstan from 2020 to 2022

**DOI:** 10.3390/v18010138

**Published:** 2026-01-21

**Authors:** Altynay Gabiden, Andrey Komissarov, Aknur Mutaliyeva, Aidar Usserbayev, Kobey Karamendin, Alexander Perederiy, Artem Fadeev, Ainagul Kuatbaeva, Dariya Jussupova, Askar Abdaliyev, Manar Smagul, Yelizaveta Khan, Marat Kumar, Temirlan Sabyrzhan, Aigerim Abdimadiyeva, Aidyn Kydyrmanov

**Affiliations:** 1 National Center of Public Health Care, The Ministry of Health of the Republic of Kazakhstan, Almaty 050008, Kazakhstan; altinai_s@mail.ru (A.G.);; 2 Smorodintsev Research Institute of Influenza, 197022 Saint Petersburg, Russia; 3 Research and Production Center for Microbiology and Virology, Almaty 050010, Kazakhstan; 4 Faculty of Natural Sciences and Geography, Abai Kazakh National Pedagogical University, Almaty 050010, Kazakhstan; 5 National Center of Expertise of the Committee of Sanitary and Epidemiological Control, The Ministry of Health of the Republic of Kazakhstan, Astana 010000, Kazakhstan

**Keywords:** SARS-CoV-2, COVID-19, next-generation sequencing (NGS), virus evolution, mutation, variants, Kazakhstan

## Abstract

Coronavirus disease 2019 (COVID-19), caused by SARS-CoV-2, has had major social and economic consequences worldwide. Whole genome sequencing (WGS) is essential for genomic monitoring, enabling tracking of viral evolution, detection of emerging variants, and identification of introductions and transmission chains to inform timely public health responses. Here, we compile and harmonize SARS-CoV-2 genomic data generated by multiple laboratories across Kazakhstan together with publicly available sequences to provide a national overview of genomic dynamics across successive epidemic waves from 2020 to 2022. We analyzed 4462 genomes deposited in GISAID (including 340 generated in this study), of which 3299 passed Nextclade quality filters, and summarized lineage turnover across major phases (pre-VOC, Alpha, Delta, Omicron BA.1/BA.2, Omicron BA.4/BA.5, and a later recombinant-dominant period). Sequencing intensity varied markedly over time (0.60‰ of confirmed cases during Delta vs. 11.57‰ during the Omicron BA.5 wave), suggesting that lineage diversity and persistence may be underestimated. Pre-VOC circulation included ≥12 Pango lineages with evidence of multiple introductions and sustained local transmission, including a Kazakhstan-restricted B.4.1 lineage that emerged in Nur-Sultan/Astana and disappeared after April 2020. The Tengizchevroil oilfield outbreak comprised B.1.1 viruses with phylogenetic support for ≥three independent introductions. Alpha and Omicron waves were characterized by repeated introductions and heterogeneous origins, whereas Delta was dominated by AY.122 with an additional distinct AY.122 cluster; a notable BF.7 local transmission event was observed during BA.5. We also highlight locally enriched non-lineage-defining mutations. Overall, recurrent importations and variable local amplification shaped SARS-CoV-2 dynamics in Kazakhstan, while interpretation is constrained by strongly time-skewed sequencing.

## 1. Introduction

The Severe Acute Respiratory Syndrome Coronavirus 2 (SARS-CoV-2) was first identified in Wuhan, China, at the end of 2019 and rapidly spread, evolving into a global pandemic [1,2]. SARS-CoV-2 is an enveloped, non-segmented, positive single-stranded RNA virus with a genome size of about 30,000 bases, featuring four structural proteins: spike (S), envelope (E), membrane (M), and nucleocapsid (N) [3,4]. Overall, SARS-CoV-2 shares approximately 79.5% and 96% genomic sequence identity with previously identified SARS-CoV and bat coronavirus, SL-CoV-RaTG13, respectively [5,6].

SARS-CoV-2 exhibits a propensity for multiple recombinations and mutations in its genome, potentially leading to changes in viral protein structure, binding affinity, virus transmission, diagnostics, vaccine efficacy, and sensitivity to antiviral drugs [7,8,9]. Since the onset of the pandemic, the SARS-CoV-2 virus has evolved into new variants of interest (VOIs) or variants of concern (VOCs). During the circulation of early virus variants such as Alpha, Beta, Delta, etc., a high mortality rate was observed among elderly individuals and those with comorbidities such as hypertension, kidney disorders, cancer, diabetes, and obesity [10]. Later variants of the virus, including the Omicron group of strains, caused less severe illness compared to previous variants but led to an increase in cases among young people and children [11].

The first confirmed case of COVID-19 in Kazakhstan was reported on 13 March 2020. This individual had traveled from Germany. The initial virus strain in Kazakhstan was the same as the original strain identified in Wuhan, China [12]. From May 2020, Kazakhstan saw an increase in cases. Early studies of the virus indicated the circulation of the original SARS-CoV-2 strain, which caused significant outbreaks around the world [13].

From the beginning of 2021, more transmissible SARS-CoV-2 variants began to spread, including the Alpha variant (B.1.1.7), first identified in the UK, and the Beta variant (B.1.351), first identified in South Africa. From July 2021 onward, Kazakhstan began to register cases caused by the Delta variant (B.1.617.2), which is characterized by higher transmissibility and partial immune evasion. Delta subsequently became the dominant variant in the country, triggering a new wave of the pandemic [14,15].

The Omicron strain was officially confirmed in Kazakhstan in January 2022. It spread quickly across the country, helped by its high R0 (reproduction rate, meaning how quickly the virus spreads). This SARS-CoV-2 variant was more contagious than previous ones, but at the same time, it caused less severe disease in most people, which led to a significant number of infections but relatively fewer severe cases [16].

In February 2022, Kazakhstan experienced a peak in cases caused by the Omicron strain. Against this background, the number of patients with COVID-19 in medical institutions increased significantly, but most cases were mild or moderate. Thus, Omicron subvariants were dominant in winter–spring 2023 [17].

From March 2022, subvariants BA.1 and BA.2 began to actively circulate. They were then joined by the BA.4 and BA.5 subvariants. These subvariants had improved immune evasion properties, which contributed to the increase in cases despite high vaccination rates and previous infections.

Thus, the purpose of this study was to analyze the dynamics of the spread of SARS-CoV-2 virus variants in Kazakhstan in the period from 2020 to 2023, as well as to identify key mutations characteristic of variants circulating in Kazakhstan.

## 2. Materials and Methods

### 2.1. Sample Collection

The Reference Laboratory for Viral Infection Control (Almaty, Kazakhstan), which is the National Influenza Center in Kazakhstan, received 10% of SARS-CoV-2-positive samples from 18 regional virology laboratories in the country for sequencing every month.

The samples used in this study were nasopharyngeal and oropharyngeal swabs selected according to the following epidemiological and clinical criteria: (1) samples from individuals with symptoms of acute respiratory viral infections and COVID-19; (2) contact with confirmed cases of COVID-19; (3) samples from individuals arriving from abroad; (4) samples from home foci of infection; (5) samples from individuals with moderate/severe disease and/or hospitalized in intensive care units, etc.

From 2022, monitoring of coronavirus infection was introduced into the current epidemiological surveillance system for acute respiratory viral infections and influenza. All samples collected within the framework of the surveillance were tested in parallel for influenza and SARS-CoV-2; during routine studies for SARS-CoV-2, some negative samples were tested for influenza.

### 2.2. Screening PCR

In regional laboratories, PCR with real-time detection for identification of SARS-CoV-2 virus RNA was performed using the Amplitest SARS-CoV-2 reagent kit (CRIE, Moscow, Russia), Intifika SARS-CoV-2 (Alkor Bio Company Ltd., Saint-Petersburg, Russia), BGI (BGI Genomics, Shenzhen, China), etc. The CDC’s Influenza SARS-CoV-2 Multiplex Assay reagent kit was used in the reference laboratory.

### 2.3. Virus Genome Sequencing

Viral RNA extraction was performed using the commercial PureLink RNA MiniKit (Life Technologies, Carlsbad, CA, USA) and QIAamp Viral RNA Mini Kit (Qiagen GmbH, Hilden, Germany) according to the manufacturers’ recommendations. Reverse transcription and SARS-CoV-2 library preparation were performed using the Ampliseq™ cDNA Synthesis and AmpliSeq Library PLUS for Illumina kits (Illumina, San Diego, CA, USA) according to the manufacturer’s recommendations. Amplification products were purified using the AMPure XP reagent (Beckman Coulter, Inc., Brea, CA, USA) according to the manufacturer’s recommendations. DNA fragment library quality was determined using the Agilent High Sensitivity DNA Kit on a Bioanalyzer 2100 (Agilent Technologies, Santa Clara, CA, USA) according to the manufacturer’s recommendations. Quantitative analysis of cDNA libraries was performed using the NEBNext Library Quant Kit for Illumina according to the manufacturer’s recommendations.

For whole genome sequencing, the MiniSeq High output Reagent Cartridge was used in PE paired-end reading mode according to the manufacturer’s recommendations. Whole genome sequencing of SARS-CoV-2 viruses was performed using the Illumina MiniSeq next-generation sequencer (NGS).

### 2.4. Bioinformatics and Phylogenetics

A sequencing quality check was performed using fastqc 0.12.1 with subsequent quality trimming using trimmomatic 0.39. Consensus genome assembly was performed using BWA v. 0.7.17, Samtools v. 1.19.2, and Ivar v.1.4.2, along with custom Python 3.10.4 scripts. All sequenced genomes were timely submitted to EpiCoV GISAID. In total, 4462 SARS-CoV-2 genomic sequences available in GISAID were downloaded and quality-filtered using Nextclade [18]. Qc.overallStatus equal to “good” or “mediocre” was used as a quality filtration criterion. A total of 3299 SARS-CoV-2 genomes from Kazakhstan passed quality filtration. First we stratified SARS-CoV-2 genomes into pandemic waves using collection dates and VOC designations: 122 genomes in the pre-VOC period, 167 in the Alpha period, 717 in the Delta period, 823 in the Omicron BA.1/BA.2 period, and 1193 in the Omicron BA.4/BA.5 period. Uvaia v2.0.1 software was used to search for the closest neighboring sequences available in GISAID (mmsa-2024-02-04.tar.xz global alignment was used as an input). Briefly, each set of SARS-CoV-2 genomes from corresponding waves was aligned to the Wuhan reference genome (EPI_ISL_402124) using uvaialign (e.g., for the Alpha wave the following command was used: uvaialign -r EPI_ISL_402124.fasta VOC_alpha.fasta). Then the uvaia search was performed as follows: “uvaia -r mmsa-2024-02-04.tar.xz/2024-02-04_masked.fa –trim=230 KZ_alpha/uvaia.10dc4_alphaKZ.aln.xz -o alpha_result --nthreads=120 -x”. Rank 1 and rank 2 neighbors were used for further analysis. Multiple alignment was performed using MAFFT software (Version 7.526) [19]. Phylogenetic tree reconstruction was conducted in RAxML-NG (“raxml-ng --msa MSA_dataset.fasta --prefix MSA_dataset --model GTR”) [20]. Treemmer tool v0.3 [21] was used to remove redundant sequences while preserving phylogenetic diversity. Final visualization of phylogenetic trees was performed using custom R scripts (v.4.4.1 ggtree package). The overall analysis workflow is presented in Supplementary Figure S1. The final list of viruses after quality filtration is provided in Supplementary Table S1.

### 2.5. Ethical Principles

In Kazakhstan, obtaining informed consent from the patient before collecting samples for COVID-19 is a mandatory procedure. During sample collection, patients filled out written voluntary consent in accordance with the order of the Ministry of Healthcare of the Republic of Kazakhstan dated 20 May 2015 No. 364 “On approval of the form of written voluntary consent of the patient for invasive interventions”. This retrospective study used anonymized residual samples obtained as part of routine and sentinel epidemiological surveillance of influenza and respiratory infections in Kazakhstan. The analysis of anonymized data and samples for scientific purposes was carried out in accordance with the Code of the Republic of Kazakhstan “On Public Health and the Healthcare System” (Article 9, Clause 4). The study was reviewed and approved by the Local Bioethics Committee of the RSE on the Right of Economic Management “National Center for Public Health” of the Ministry of Health of the Republic of Kazakhstan (Protocol No. 1 dated 30 April 2021) and the Local Ethics Committee of the Research and Production Center of Microbiology and Virology (Protocol No. 17 dated 30 October 2023).

## 3. Results

**Figure 1:**
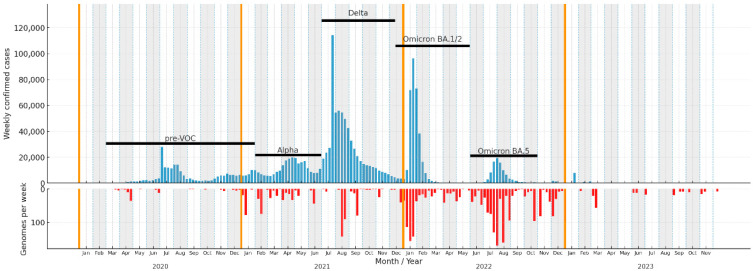
COVID-19 weekly new cases in Kazakhstan, epidemic waves, and number of sequenced genomes (in red).

Kazakhstan experienced five COVID-19 waves between March 2020 (first confirmed SARS-CoV-2 case) and the end of the reporting period in late 2022. For analysis, we stratified the timeline by epidemic waves and the emergence of WHO variants of concern (VOCs) in Kazakhstan: (i) pre-VOC—from March 2020 to January 2021 (first detection of Alpha/B.1.1.7); (ii) Alpha period—February 2021 to June 2021 (until first detection of Delta/B.1.617.2); (iii) Delta period—July 2021 to December 2021 (until first detection of Omicron BA.1/BA.2); (iv) Omicron period, further subdivided into BA.1/BA.2 and BA.4/BA.5 sub-periods, followed by a recombinant-dominant phase (Figure 1).

A total of 4462 SARS-CoV-2 genomes were obtained from GISAID, of which 340 were generated in this study. Following quality assessment with Nextclade, 3299 genomes met high- or medium-quality thresholds and were retained for downstream analyses.

Genomic sequencing was unevenly distributed over time, with scarce data early in the pandemic and a marked increase in the second and third years (Table 1). The proportion of confirmed cases sequenced ranged from 0.60‰ (per mille) during the Delta wave—when case counts were high and sequencing capacity limited—to 11.57‰ during the Omicron BA.5 wave. After the WHO ended the COVID-19 Public Health Emergency of International Concern on 5 May 2023, case registration practices changed substantially; therefore, we did not calculate sequencing proportions for the subsequent period.

**Table 1:** Proportion of sequenced genomes during different periods of COVID-19 pandemic in Kazakhstan.

Period	Start	End	Total Cases	Total Genomes	Proportion Sequenced, ‰	95% CI
pre-VOC	2020-03-01	2021-01-31	227,165	193	0.85	0.74–0.98
Alpha	2021-02-01	2021-06-30	250,898	328	1.31	1.17–1.46
Delta	2021-07-01	2021-12-14	645,121	387	0.60	0.54–0.66
Omicron BA.1/BA.2	2021-12-15	2022-05-31	331,058	772	2.33	2.17–2.50
Omicron BA.5	2022-06-01	2022-10-31	91,081	1054	11.57	10.90–12.29

### 3.1. Genetic Diversity of SARS-CoV-2 Circulated in Kazakhstan in Pre-VOC Period (March 2020–January 2021)

**Figure 2:**
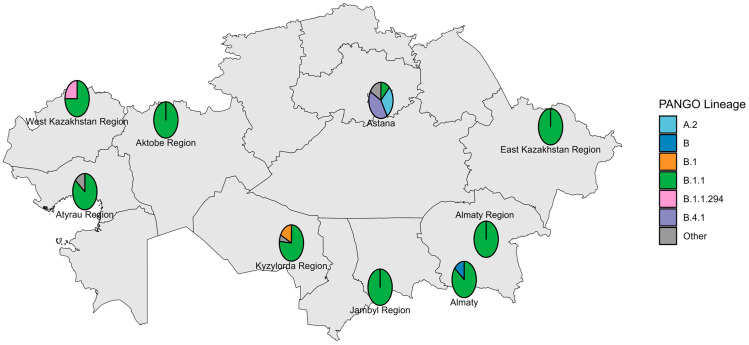
Geographical distribution of sequenced SARS-CoV-2 genomes in the pre-VOC period.

**Figure 3:**
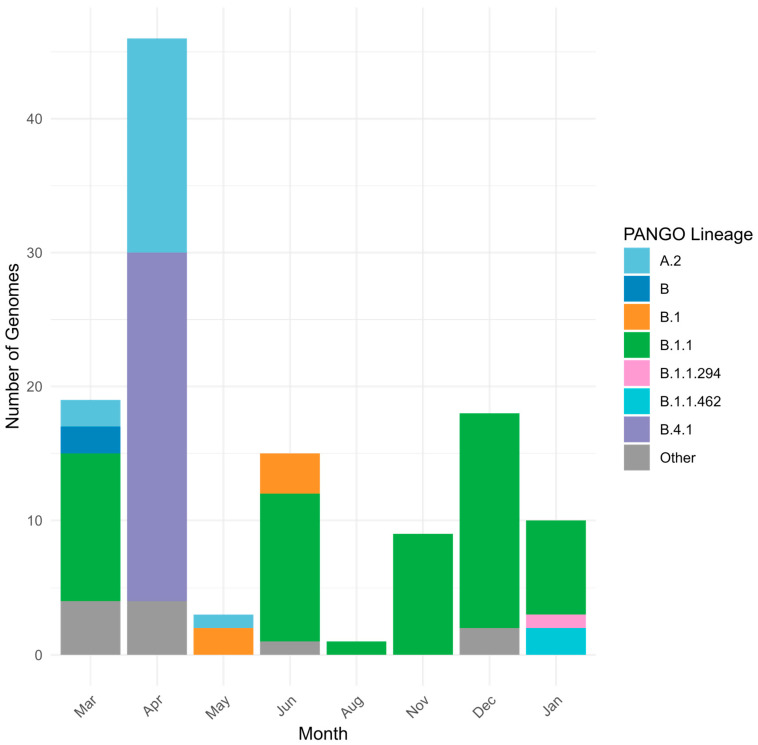
Genetic diversity of SARS-CoV-2 in Kazakhstan in pre-VOC period (March 2020–January 2021).

The first COVID-19 case in Kazakhstan was identified in Almaty on 13 March 2020. The earliest genomic data were generated in May 2020 from specimens collected in Nur-Sultan (now Astana) in late March 2020, with most early strains sequenced retrospectively. During the pre-VOC period, 12 Pango lineages circulated nationwide; B.1.1 predominated across most locations and months, whereas Astana was dominated by B.4.1 (Figure 2 and Figure 3).

Phylogenetic analysis of Kazakhstan samples from the pre-VOC period resolved six clades corresponding to A, B, A.2, B.1, B.1.1, and B.4.1. Within B.1.1, we observed diversification into B.1.1.10, B.1.1.294, B.1.1.336, B.1.1.440, and B.1.1.462. Nearest-neighbor clustering with Uvaia against a global background indicated multiple, sustained local transmission chains during the first epidemic wave of COVID-19 in Kazakhstan.

Geographically, A and B lineage viruses grouped with contemporaneous sequences from China and Malaysia, whereas A.2 grouped with European sequences (notably Spain and Portugal). B.1 sequences predominantly grouped with Asian sequences (Saudi Arabia and Indonesia), with a minor cluster linked to Iceland.

Lineage B.4.1 formed a distinct, Kazakhstan-only cluster. The sequenced B.4.1 genome was collected in Nur-Sultan (now Astana) in late March 2020. Its putative ancestor, B.4, was detected in Wuhan in January 2020 and became highly prevalent in Iran (303/740 B.4 sequences in GISAID). Compared with B.4, the Kazakhstan B.4.1 lineage carries ORF1ab substitutions I1023K and A1225D, plus a deletion at position 1024. B.4.1 forms a monophyletic clade restricted to Nur-Sultan, with no phylogenetic evidence of exportation and no circulation observed after April 2020.

B.1.1.294 was first reported in Russia in March 2020. In Kazakhstan, B.1.1.294 was detected in December 2020–January 2021 in the West Kazakhstan region and Nur-Sultan (Astana). The highest relative prevalence in the region was observed in Uzbekistan, where 4/28 genomes sequenced between March 2020 and January 2021 belonged to B.1.1.294. Phylogenetic analysis clustered Kazakhstan B.1.1.294 sequences with contemporaneous viruses from Russia and South Korea. According to GISAID metadata, 7/13 B.1.1.294 cases reported in South Korea were imports (six independent introductions from Uzbekistan during July–October 2020 and one from Russia in September 2020). Because genomic surveillance in Central Asia was limited in 2020, the origin of B.1.1.294 remains uncertain; a Russian source is plausible but cannot be determined with confidence.

B.1.1.440 was a predominantly U.S.-centered pre-VOC lineage: 89/93 available genomes were collected in the United States or U.S. territories, including 86 from Texas. Two sequences from Baikonur (Kazakhstan) cluster phylogenetically with a South Korean genome (hCoV-19/South_Korea/KDCA3546/2020) that GISAID metadata annotate as an export from Kazakhstan to South Korea. The Baikonur sequences appear closely related to two genomes from the Northern Mariana Islands.

**Figure 4:**
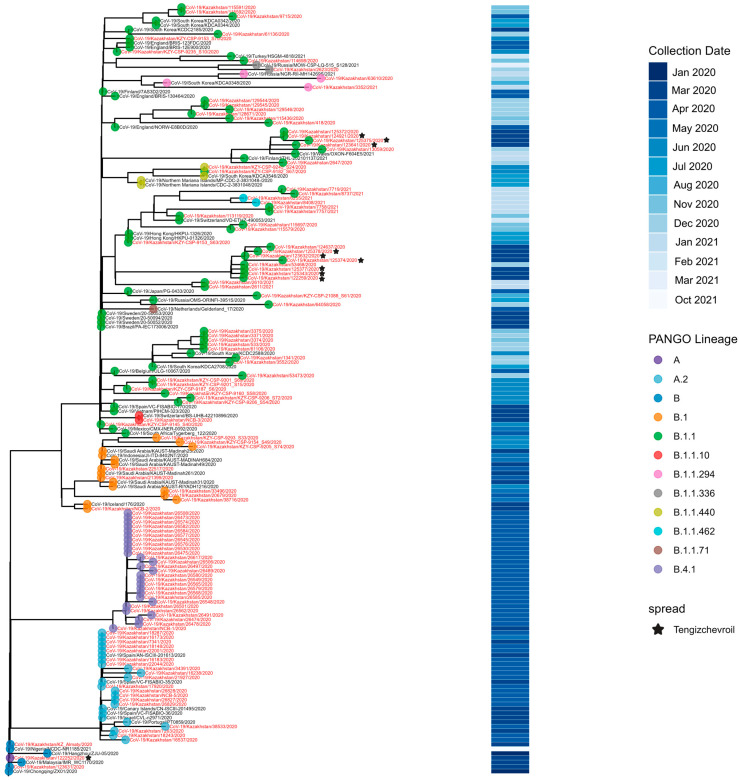
Phylogenetic analysis of SARS-CoV-2 in Kazakhstan in pre-VOC period.

Tengizchevroil (TCO), in the Atyrau Region, became Kazakhstan’s largest workplace COVID-19 hotspot in 2020. Between March and May 2020, 1306 laboratory-confirmed infections among workers were recorded [22]. In May 2020, as the Ministry of Health considered suspending operations, TCO demobilized ~20,000 of its ~30,000 staff and instituted pre-rotation quarantine with PCR testing for the remaining ~13,000 employees. Despite these measures, cumulative infections rose to 2661 by 29 July 2020 [22]. Genomic data from the outbreak indicate that all sequenced cases belonged to Pango lineage B.1.1. Phylogenetic reconstruction supports at least three independent introductions of SARS-CoV-2 into the Tengiz oilfields (Figure 4).

### 3.2. Genetic Diversity of SARS-CoV-2 in Kazakhstan in Alpha Period (February–June 2021)

**Figure 5:**
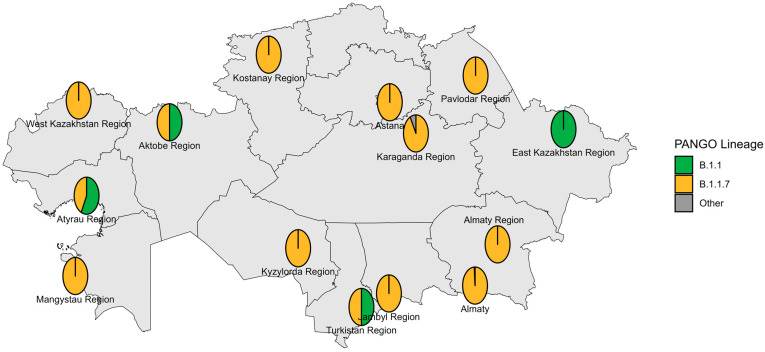
Geographical distribution of sequenced SARS-CoV-2 genomes in the Alpha period.

**Figure 6:**
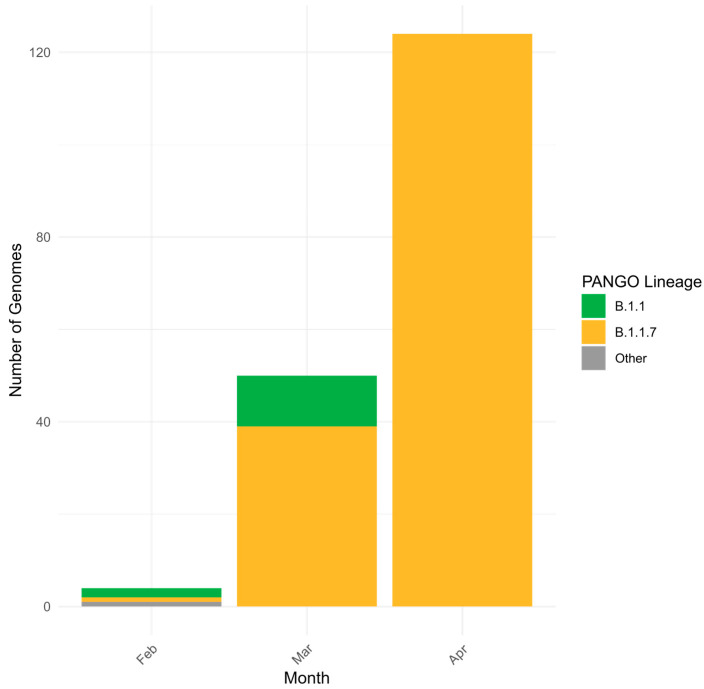
Genetic diversity of SARS-CoV-2 in Kazakhstan in Alpha period (February–April 2021).

Sequenced Alpha VOC viruses had wide geographical distribution (Figure 5). The first Alpha variant viruses were reported in Kazakhstan at the end of March 2021. Retrospective sequencing revealed the oldest specimen positive for Alpha to be from the beginning of February 2021 [23]. Most of the sequenced specimens were collected in March and April 2021 (Figure 6).

The B.1.1.7 lineage harbors eighteen amino acid changes compared with the Wuhan-Hu-1 reference—four of which are shared with its parental B.1.1 lineage—along with three in-frame deletions (ORF1a:del3676/3678, S:del69/70, and S:del144). In addition to the lineage-defining set, nine nonsynonymous mutations occurred in at least 10% of Kazakhstan viruses (Table 2), with several showing higher prevalence in Kazakhstan than globally.

Several mutations in the Kazakhstan Alpha dataset show markedly higher frequencies than in global datasets, suggesting potential regional enrichment or distinct local transmission dynamics. The most striking enrichment is observed for ORF1b I28T (NSP12:I37T), detected in 69/167 (41.32%) genomes from Kazakhstan yet virtually absent in global Alpha datasets (0.01%). Additional recurrent changes include ORF1a F200L (NSP13:F200L) in 28/167 (16.77%) and ORF7a P84L (NS7a:P84L) in 30/167 (17.96%), both of which were rare worldwide (0.00% and 0.28%, respectively).

Several substitutions enriched in Kazakhstan reached approximately 10–11% locally: M F100I (M:F100I) and ORF1a M1312I (NSP3:M494I) (each 19/167; 11.38%), both essentially absent globally (0.00% and 0.08%), and ORF3a Y189S (NS3:Y189S), ORF3a A99S (NS3:A99S), and ORF1a V3595D (NSP6:V26D) (each 18/167; 10.78%), all rare worldwide (0.01%, 0.06%, and 0.00%, respectively). In contrast, ORF8 Y73C (NS8:Y73C) was highly frequent both in Kazakhstan (115/167; 68.86%) and globally (94.93%), representing a characteristic Alpha mutation rather than a region-specific variant. Similarly, ORF8 K68stop (NS8:K68stop) appeared at a lower frequency in Kazakhstan (23/167; 13.77%) compared with global datasets (33.23%).

**Figure 7:**
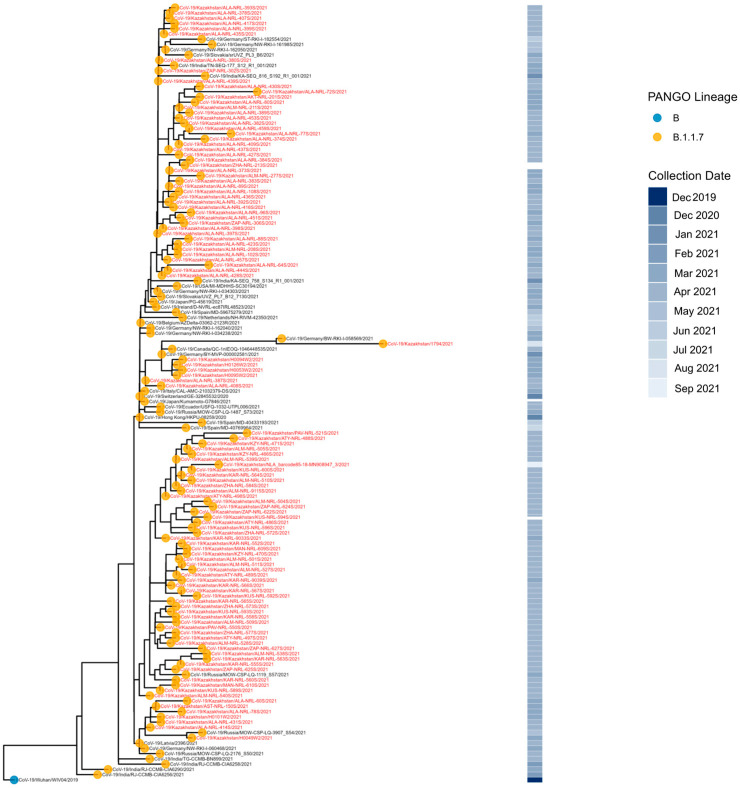
Phylogenetic analysis of Alpha VOC SARS-CoV-2 in Kazakhstan.

The placement of Kazakhstan B.1.1.7 sequences across the phylogeny (Figure 7) indicates multiple introductions, as they do not form a monophyletic group. Notably, many appear as singletons or in small clades, suggesting that most introductions did not result in sustained community transmission

### 3.3. Genetic Diversity of SARS-CoV-2 in Kazakhstan in Delta Period (July–December 2021)

**Figure 8:**
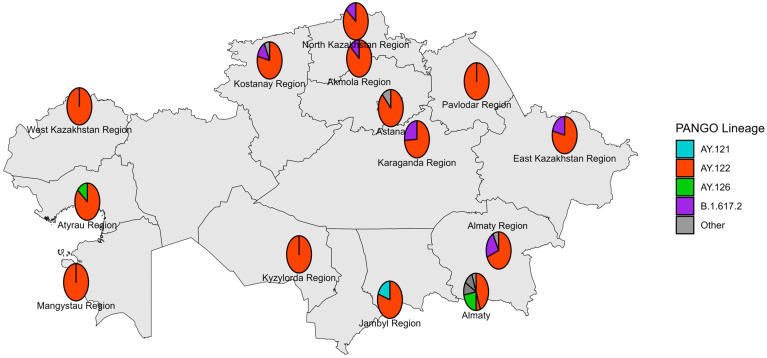
Geographical distribution of sequenced SARS-CoV-2 genomes in the Delta period.

**Figure 9:**
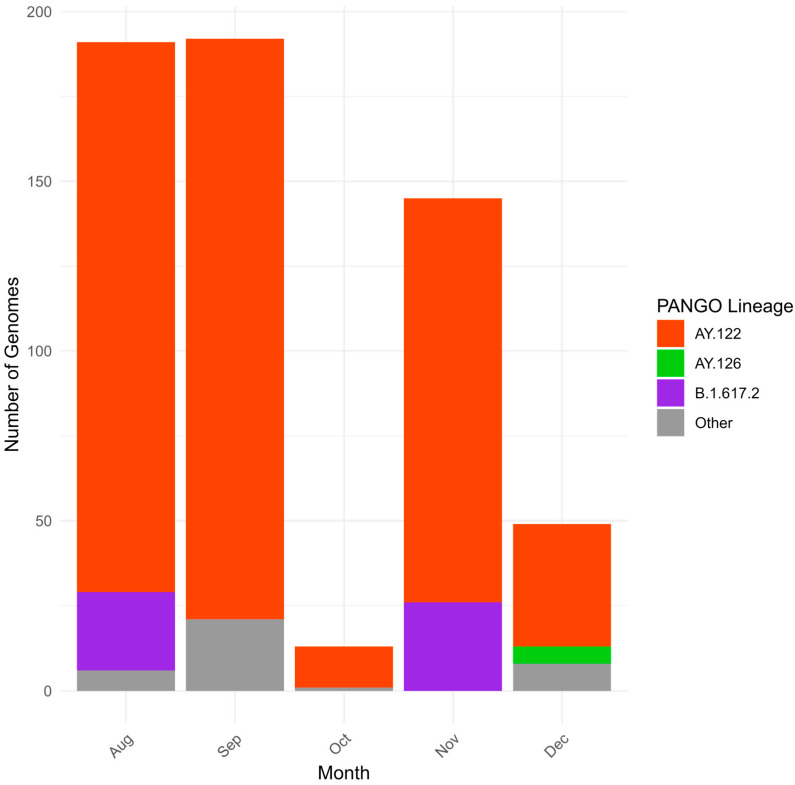
Genetic diversity of SARS-CoV-2 in Kazakhstan in Delta period (August–December 2021).

**Figure 10:**
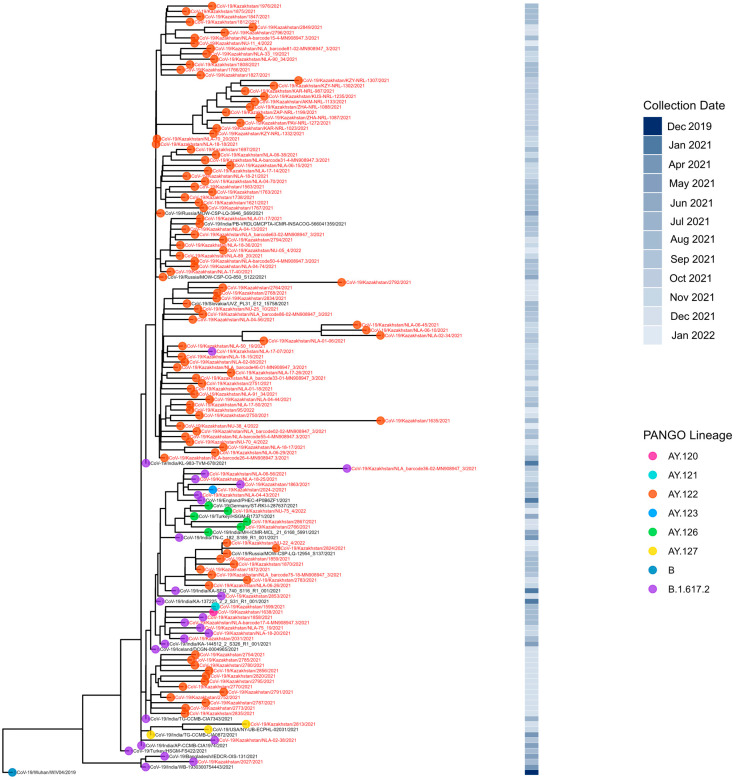
Phylogenetic analysis of Delta VOC SARS-CoV-2 in Kazakhstan.

The first Delta strain cases were identified in Kazakhstan in July 2021 [24], yet reliable genomic data start from August 2021. Sequenced Delta VOC viruses had wide geographical distribution (Figure 8). Most of the sequenced specimens were collected in August, September, and November 2021 (Figure 9). The dominating lineage was AY.122 with combination of NS7a_P45L and NSP2_K81N substitutions typical for the previously described Russian sublineage of AY.122 [19]. Interestingly, a monophyletic cluster of AY.122 viruses without NS7a_P45L+ NSP2_K81N combination typical for Russian AY.122 viruses grouping with a virus of Indian origin was observed (Figure 10). Due to the low sequencing level in Russia and Central Asia at that time, it is hard to locate the plausible place of emergence of these AY.122 sublineages and the directions of their importation/exportation; nevertheless, we can speculate that AY.122 in Kazakhstan was not as homogeneous as AY.122 in Russia. The sporadic detection of AY.120, AY.121, AY.123, AY.126, and AY.127 lineages was observed.

The most characteristic Delta variant mutations showed near-universal prevalence in Kazakhstan sequences: Spike D950N in 606/613 (98.86%) and NSP14 A394V in 520/613 (84.83%), both of which were also highly conserved globally (90.63% and 98.68%, respectively). Additional defining Delta mutations included Spike T478K in 553/613 (90.21%), Spike L452R in 545/613 (88.91%), and Spike G142D in 566/613 (92.33%), all with similarly high global frequencies (97.11%, 96.83%, and 60.53%, respectively).

The NS7a substitutions P45L, T120I, and V82A were detected in 509/613 (83.03%), 487/613 (79.45%), and 507/613 (82.71%) of Kazakhstan sequences, with corresponding global frequencies of 62.89%, 93.43%, and 91.53% (Table 3). These represent characteristic AY.122 lineage markers rather than Kazakhstan-specific variants. In contrast, NSP3 H1841Y showed notable enrichment in Kazakhstan, occurring in 56/613 (9.14%) sequences compared with only 0.30% globally, suggesting potential regional selection or distinct local transmission dynamics within the Kazakhstan SARS-CoV-2 Delta variant population.

**Table 3:** Frequency of additional AY.122 substitutions observed in sequences from Kazakhstan.

Gene	Substitution	GISAID Notation	Number in Dataset	Frequency in Dataset	Number in Global Dataset	Frequency in Global Dataset
NS7a	P45L	NS7a_P45L	509	83.03	154,034	62.89
NS7a	T120I	NS7a_T120I	487	79.45	228,836	93.43
NS7a	V82A	NS7a_V82A	507	82.71	224,181	91.53
NSP14	A394V	NSP14_A394V	520	84.83	241,699	98.68
NSP3	H1841Y	NSP3_H1841Y	56	9.14	745	0.30
Spike	G142D	Spike_G142D	566	92.33	148,264	60.53
Spike	D950N	Spike_D950N	606	98.86	221,980	90.63
Spike	L452R	Spike_L452R	545	88.91	237,174	96.83
Spike	T478K	Spike_T478K	553	90.21	237,850	97.11

### 3.4. Genetic Diversity of SARS-CoV-2 Circulated in Kazakhstan in Omicron BA.1/BA.2 Period (January–May 2022)

**Figure 11:**
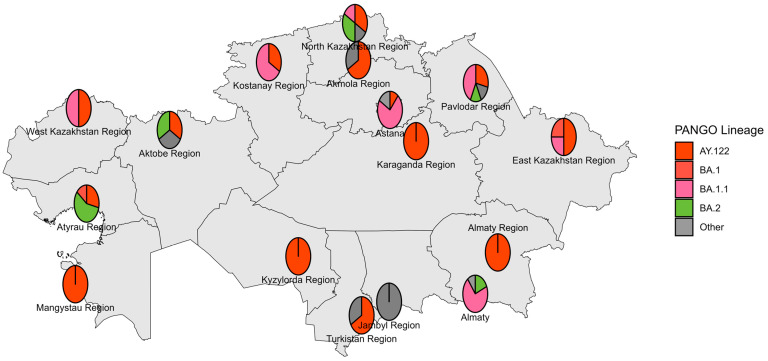
Geographical distribution of sequenced SARS-CoV-2 genomes in the Omicron BA.1/BA.2 period.

**Figure 12:**
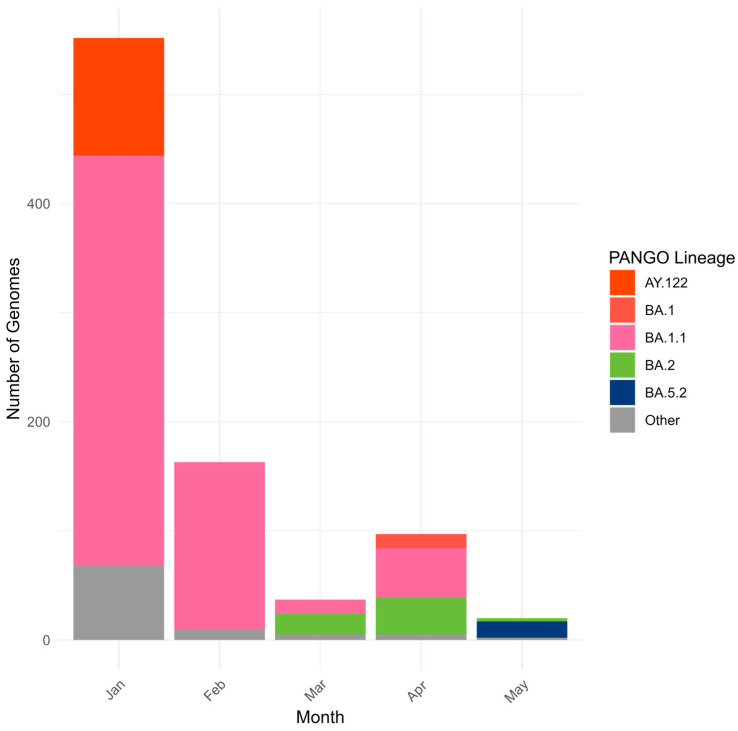
Genetic diversity of SARS-CoV-2 in Kazakhstan in Omicron BA.1/BA.2 period (January–May 2022).

**Figure 13:**
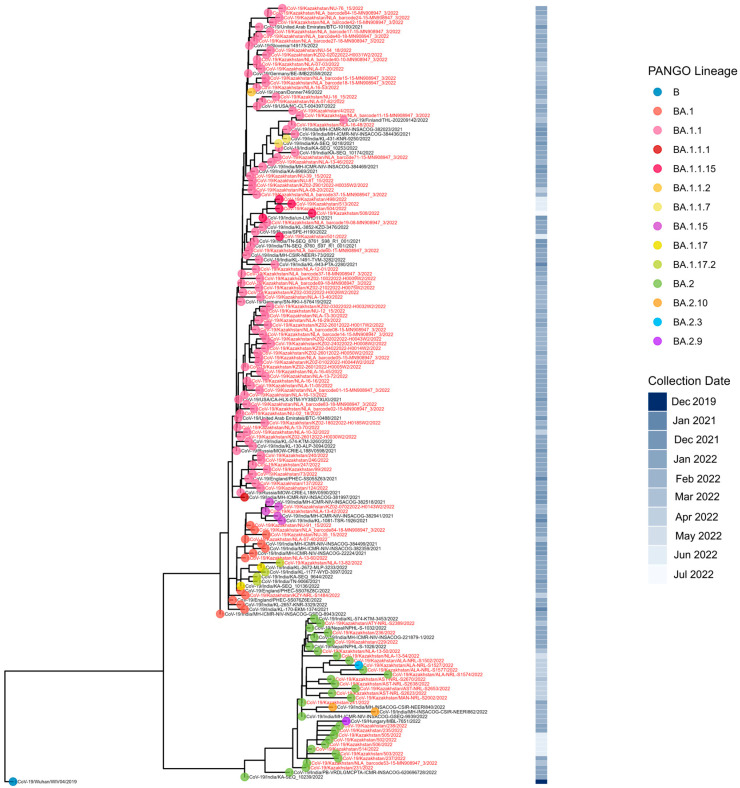
Phylogenetic analysis of Omicron BA.1/BA.2 SARS-CoV-2 in Kazakhstan.

The first Omicron case was reported in Kazakhstan in January 2022, followed by a high epidemic wave and a drastic decline within less than a month. Sequenced BA.1/BA.2 viruses showed a wide geographic distribution but a strongly skewed temporal distribution: more than 50% of sequenced specimens were collected in January 2022, at the start of the Omicron BA.1/BA.2 wave in Kazakhstan (Figure 11 and Figure 12). The dominant lineage was BA.1 and its sublineages. The most prevalent BA.1.1 did not form a monophyletic cluster, with many branches of the phylogenetic tree clustering with Indian SARS-CoV-2 genomes (Figure 13). It is interesting to note that inbound tourism from India to Kazakhstan constantly grew from 1603 visitors in 2021 to 10,090 visitors in 2022 [25]. International air travel was resumed in Kazakhstan in September 2021. BA.2 genomes from Kazakhstan also clustered with SARS-CoV-2 viruses collected in India and Nepal.

### 3.5. Genetic Diversity of SARS-CoV-2 Circulated in Kazakhstan in Omicron BA.4/BA.5 Period (June–October 2022)

**Figure 14:**
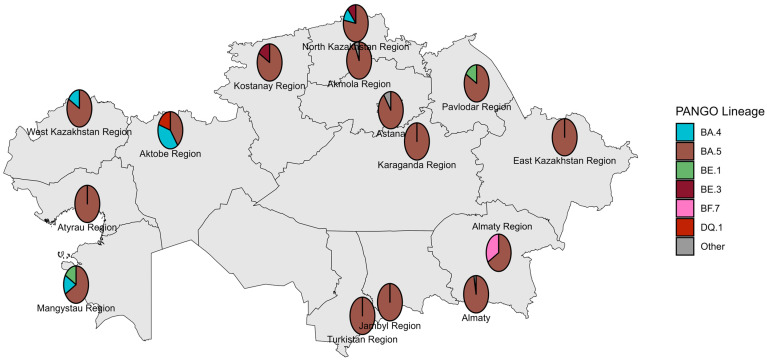
Geographical distribution of sequenced SARS-CoV-2 genomes in the Omicron BA.4/BA.5 period.

**Figure 15:**
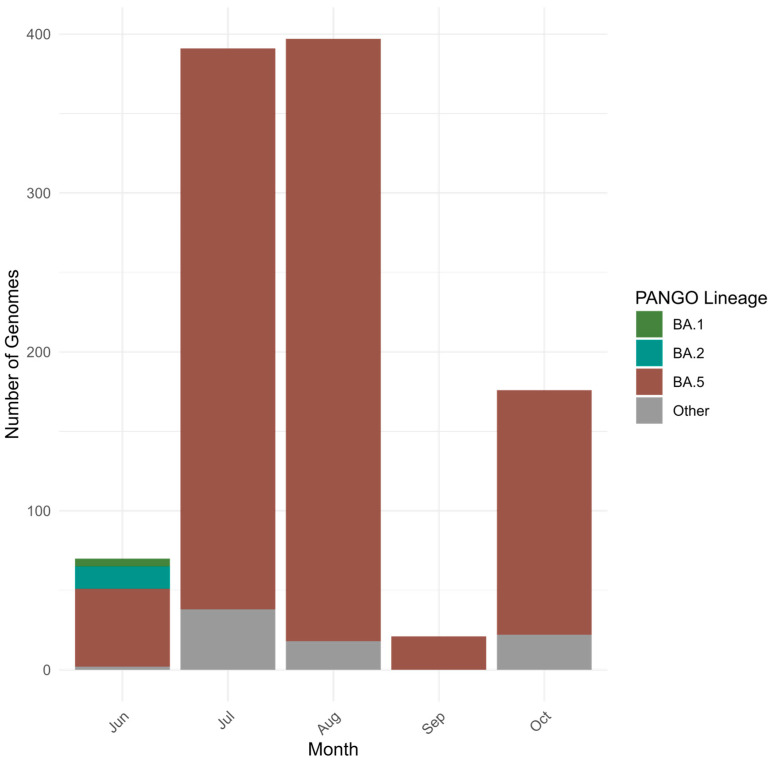
Genetic diversity of SARS-CoV-2 in Kazakhstan in Omicron BA.4/BA.5 period (June–October 2022).

**Figure 16:**
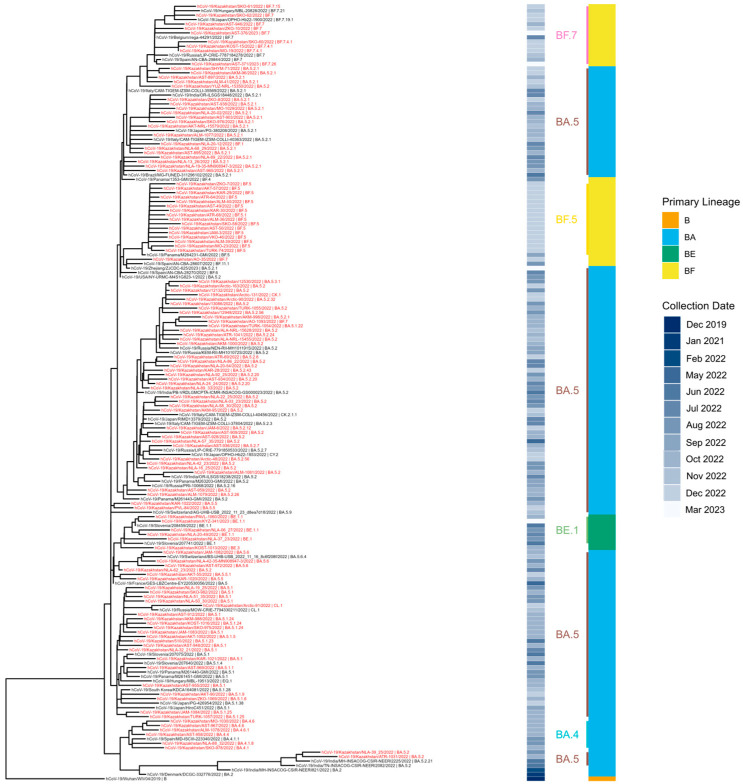
Phylogenetic analysis of Omicron BA.4/BA.5 SARS-CoV-2 in Kazakhstan.

The earliest Omicron BA.4/BA.5 genomes in Kazakhstan were collected in June 2022. Most sequenced specimens, however, were collected in August and September 2022 (Figure 14 and Figure 15). BA.5 and its sublineages predominated. Among BA.5 descendants, the most prevalent were BA.5.2, BA.5.1, BF.5 (BA.5.2.1.5), BF.7 (BA.5.2.1.7), and BE.1 (BA.5.3.1.1). Phylogenetic clustering with the closest global sequences suggested a large local transmission event involving the BF.7 lineage, affecting many regions of Kazakhstan, with the nearest non-Kazakhstan BF.7 genome in the tree originating from Central America (Figure 16). In contrast, BE.1 sequences from Kazakhstan clustered mainly with European genomes (notably from Slovenia). BA.5.2 strains appeared to be of heterogeneous origin, with multiple introductions forming clusters closely related to Russian, Indian, and various European sequences. BA.4 genomes from Kazakhstan clustered with European SARS-CoV-2 sequences.

## 4. Discussion

Kazakhstan experienced five COVID-19 waves from March 2020 through 2023. We analyzed 3299 quality-filtered genomes (4462 from GISAID; 340 newly generated), stratifying by epidemic phase—pre-VOC (March 2020–9 February 2021), Alpha (9 February –June 2021), Delta (July–December 2021), Omicron BA.1/BA.2 (December 2021–May 2022), and Omicron BA.4/BA.5 (June–October 2022)—providing new insights into the spread of SARS-CoV-2 in Central Asia. At the same time, the very uneven sequencing intensity across waves (from 0.60‰ of cases during Delta to 11.57‰ during the Omicron BA.5 period) means that inferences about relative lineage diversity and persistence must be interpreted with caution, as the diversity of SARS-CoV-2 viruses in Kazakhstan is likely undersampled.

The pre-VOC phase in Kazakhstan was characterized by the co-circulation of at least 12 Pango lineages across six major clades (A, B, A.2, B.1, B.1.1, and B.4.1), mirroring the broad early diversity reported previously and consistent with multiple importations from both Asian and European/American sources [25]. The explicit association of some B.1.1 sublineages with the Tengiz oilfield outbreak is epidemiologically plausible: that complex has been documented as a major national hotspot in 2020, with >1000 infections among oilfield workers in early 2020 and strong evidence for intense workplace-associated transmission [22].

The strong concentration of B.1.1.440 in Houston, Texas (home to NASA’s Johnson Space Center), together with its apparent phylogenetic connection to Baikonur (a major spaceport) via the Northern Mariana Islands (which host space-launch facilities), may reflect travel associated with space-launch operations. However, this interpretation remains speculative and cannot be confirmed with the available data.

Earlier work suggested that B.4.1 may have arisen independently in Kazakhstan; the Kazakhstan-restricted monophyletic clade that disappeared after April 2020 provides strong support for this and illustrates how geographically constrained lineages can emerge and then go extinct without contributing to the later global VOC landscape. Similar short-lived local lineages have been described elsewhere and are generally interpreted as the product of founder effects and transient ecological opportunities, which are then overwhelmed by fitter variants or by changes in mobility and control measures.

During the Alpha period, B.1.1.7’s widespread geographic presence but scattered phylogenetic placement—dominated by singletons and small clades rather than one or two large monophyletic clusters—indicates that Kazakhstan experienced numerous Alpha introductions, most of which failed to generate large, sustained community transmission chains. This pattern closely parallels detailed B.1.1.7 analyses from Denmark [26], Mexico [27], and wastewater-based studies in Europe, where repeated introductions were common but only a subset of lineages achieved major expansion. The strong local enrichment of ORF1b I28T (NSP12:I37T) among Kazakhstan Alpha genomes, despite its rarity globally, likely reflects a combination of founder effects and expansion of a particular B.1.1.7 sublineage rather than clear adaptive change.

The observed Delta period in Kazakhstan fits well into the global picture of rapid Delta replacement in mid-2021 but with some striking regional nuances. Delta became globally dominant by mid-2021, accounting for nearly all sequenced infections by late August, and similar timing has been reported across Europe and the Middle East [28].

The dominance of AY.122 carrying the nsp2:K81N and ORF7a:P45L substitutions closely parallels the situation in Russia, where >90% of Delta sequences shared this mutation pair and were assigned to AY.122, a combination that remained rare in most other countries. Klink et al. also noted that Kazakhstan was among the few settings outside Russia with a high frequency of this AY.122 signature, suggesting intense epidemiological connectivity across the region during the Delta wave. The broad geographic distribution of these AY.122 viruses within Kazakhstan allows the speculation of a scenario in which one or a few successful introduction(s) of the “Russian-like” AY.122 sublineage were amplified by sustained community transmission. At the same time, the identification of a monophyletic AY.122 cluster lacking the canonical nsp2:K81N + ORF7a:P45L combination and grouping phylogenetically with a virus of Indian origin indicates that AY.122 circulation in Kazakhstan was not genetically homogeneous. Rather, it likely reflects at least two epidemiologically distinct AY.122 sources—a Russian-linked K81N+P45L sublineage and a second introduction related to an unknown source. Similar coexistence of multiple AY.* sublineages arising from separate importation events has been documented in England and other well-sampled settings, where some sublineages expand locally while others remain confined to small clusters [29].

The BA.1/BA.2 Omicron wave in Kazakhstan, first detected in January 2022, was short but intense, with a rapid rise and decline in reported cases, consistent with the high intrinsic transmissibility and immune escape properties of BA.1/BA.2 observed globally. The genomic data show that BA.1 and its sublineages, particularly BA.1.1, dominated this wave and were widely distributed across the country, but sequencing was heavily concentrated in January, which likely captures the early expansion phase while under-representing subsequent transmission. The fact that Kazakhstan BA.1.1 genomes do not form a single monophyletic clade and instead intersperse with multiple Indian sequences, together with BA.2 genomes clustering with viruses from India and Nepal, supports a scenario of repeated Omicron introductions from South Asia rather than expansion of a single local founder lineage. The resumption of international air travel in late 2021 and the marked increase in inbound tourism from India provide plausible human mobility pathways for these introductions. However, uneven temporal sampling and limited sequencing depth constrain precise reconstruction of introduction routes and onward spread.

Overall, these results show that Kazakhstan’s epidemic was shaped by repeated international introductions, workplace and community amplification of a subset of those importations, and the rapid turnover of locally restricted lineages by globally successful VOCs. Sustained, more uniform genomic surveillance across regions and epidemic phases would not only improve reconstruction of past transmission dynamics in Kazakhstan but also strengthen the country’s ability to detect and characterize future variants of concern.

## Figures and Tables

**Table 2:** Frequency of additional B.1.1.7 substitutions observed in sequences from Kazakhstan.

Gene	Substitution	GISAID Notation	Number in Dataset	Frequency in Dataset	Number in Global Dataset	Frequency in Global Dataset
M	F100I	M_F100I	19	11.38	23	0.00
NS3	Y189S	NS3_Y189S	18	10.78	74	0.01
NS3	A99S	NS3_A99S	18	10.78	755	0.06
NS7a	P84L	NS7a_P84L	30	17.96	3372	0.28
NS8	Y73C	NS8_Y73C	115	68.86	1,137,603	94.93
NS8	K68stop	NS8_K68	23	13.77	398,194	33.23
NSP12	I37T	NSP12_I37T	69	41.32	84	0.01
NSP12	P227L	NSP12_P227L	1	0.60	173,259	14.46
NSP13	F200L	NSP13_F200L	28	16.77	48	0.00
NSP3	M494I	NSP3_M494I	19	11.38	945	0.08
NSP6	V26D	NSP6_V26D	18	10.78	19	0.00

## Data Availability

All sequences were submitted to EpiCov GISAID (EPI_SET_251114au. DOI: https://doi.org/10.55876/gis8.251114au accessed on 12 November 2025). Programming scripts used for data analysis and visualization are available at https://github.com/LMV-NIC-St-Petersburg/sars-cov-2-kazakhstan-data (accessed on 12 November 2025).

## Supplementary Materials

The following supporting information can be downloaded at https://www.mdpi.com/article/10.3390/v18010138/s1, Figure S1: Kazakhstan SARS-CoV-2 genomic dataset analysis workflow, Table S1: SARS-CoV-2 genomes from Kazakhstan after quality filtration.
