# Supplementary figures and images for: Genetic Diversity of SARS-CoV-2 in Kazakhstan from 2020 to 2022
Source: Viruses. 2026 Jan 21;18(1):138. doi: 10.3390/v18010138 (PMC12846377; doi:10.3390/v18010138)

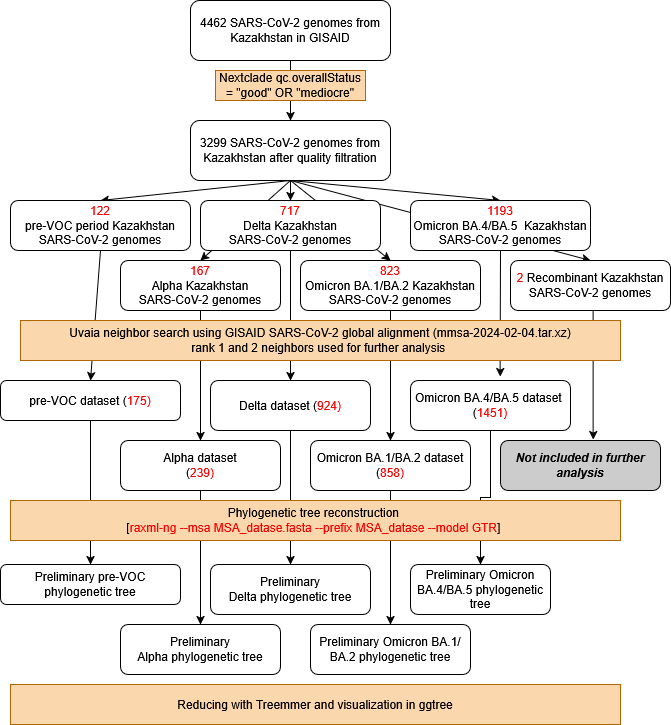

Supplement: Supplementary file 1 [file viruses-18-00138-s001.zip › FigureS1.png]
